# Analysis of distribution of IL13 and ADAM33 gene haplotypes in Russians, Khakass, Tuvans in relation to the risk of developing bronchial asthma

**DOI:** 10.18699/vjgb-26-79

**Published:** 2026-09

**Authors:** M.V. Smolnikova, K.V. Afonicheva, A.G. Mileyko

**Affiliations:** Scientific Research Institute of Medical Problems of the North – a separate division of the Federal Research Center “Krasnoyarsk Science Center” of the Siberian Branch of the Russian Academy of Sciences, Krasnoyarsk, Russia; Scientific Research Institute of Medical Problems of the North – a separate division of the Federal Research Center “Krasnoyarsk Science Center” of the Siberian Branch of the Russian Academy of Sciences, Krasnoyarsk, Russia; Scientific Research Institute of Medical Problems of the North – a separate division of the Federal Research Center “Krasnoyarsk Science Center” of the Siberian Branch of the Russian Academy of Sciences, Krasnoyarsk, Russia

**Keywords:** bronchial asthma, gene polymorphism, haplotypes, populations, IL13, ADAM33, бронхиальная астма, генетический полиморфизм, гаплотипы, популяции, IL13, ADAM33

## Abstract

Bronchial asthma (BA) is a heterogeneous disease, the development of which is determined by a complex interaction of genetic factors and environmental factors. Despite significant progress in understanding the molecular mechanisms of the disease, population-specific genetic characteristics, especially in multi-ethnic regions of Russia, remain understudied. The relevance of this study is determined by the need to identify ethnicity-specific genetic markers to improve approaches for personalized prediction of the risk of developing BA and its severity. The aim of the work was to conduct a comparative analysis of haplotypic combinations in the IL13 (rs20541, rs1800925, rs1295686) and ADAM33 (rs2280090, rs2787094, rs44707) genes between ethnic groups to identify potential population-specific genetic markers at BA-associated loci. The study involved 524 adolescents aged 12 to 18 years from Krasnoyarsk, Kyzyl and Abakan with verified ethnicity (Slavs – 293, Tuvans – 158, Khakass – 73). DNA was isolated from saliva samples followed by genotyping of polymorphic loci of the IL13 (rs20541, rs1800925, rs1295686) and ADAM33 (rs2280090, rs2787094, rs44707) genes using real-time PCR. It was found that rare variants of IL13 polymorphisms were more common in Russians than in Tuvans and the Khakass. The ATC haplotype of IL13 is 2–3 times more common among the Khakass and Tuvans than in Russians. In the Russian population, the GAC haplotype of the ADAM33 gene, containing rare alleles of all the polymorphisms studied, is detected significantly more often. The data obtained emphasize the relevance of haplotype analysis and the importance of taking into account ethno-geographical features when assessing the genetic risks of BA.

## Abstract (ru)

Несмотря на значительные успехи в исследованиях молекулярных механизмов бронхиальной астмы (БА), остаются малоизученными популяционно-генетические особенности, особенно в мультиэтнических регионах России. Актуальность изучения патогенеза астмы определяется необходимостью выявления этноспецифичных генетических маркеров для совершенствования подходов к персонифицированному прогнозированию риска развития и тяжести течения БА. Целью работы был сравнительный анализ гаплотипических комбинаций в генах IL13 (rs20541, rs1800925, rs1295686) и ADAM33 (rs2280090, rs2787094, rs44707) между этническими группами для выявления потенциальных популяционно-специфичных генетических маркеров в локусах, ассоциированных с БА. В исследовании приняли участие 524 подростка в возрасте от 12 до 18 лет из Красноярска, Кызыла и Абакана, с верифицированной национальной принадлежностью (русские – 293, тувинцы – 158, хакасы – 73). ДНК выделяли из образцов слюны с последующим генотипированием полиморфных локусов генов IL13 (rs20541, rs1800925, rs1295686) и ADAM33 (rs2280090, rs2787094, rs44707) методом ПЦР в реальном времени. Установлено, что у русских редкие варианты полиморфизмов IL13 встречаются чаще по сравнению с тувинцами и хакасами. У хакасов и тувинцев в два-три раза чаще относительно русских встречается гаплотип ATC IL13. В русской популяции значимо чаще выявлялся гаплотип GAC гена ADAM33, содержащий редкие аллели всех исследованных полиморфизмов. Полученные данные подчеркивают актуальность анализа гаплотипов и важность учета этногеографических особенностей при оценке генетических рисков развития БА.

## Introduction

Genetic predisposition, influenced by environmental factors, underlies the development of bronchial asthma (BA). The heterogeneity of BA is manifested in various endotypes (atopic, non-atopic, aspirin-induced, obesity-associated, severe steroiddependent, steroid-insensitive eosinophilic, neutrophilic, eosinophilic, etc.), variability in response to drug therapy, and multiple pathogenetic mechanisms.

According to the Russian Ministry of Health, a high prevalence of asthma was revealed by epidemiological data. In 2019, there were more than 350 million patients worldwide, with children comprising 10 %. In the Russian Federation, 1.591 million patients were diagnosed with asthma in 2022, including 84,000 adolescents aged 15–17 years and 229,000 children aged 0–14 years. In 2019, the overall incidence of asthma in the Siberian Federal District exceeded the Russian average (1,507.1 per 100,000 total population) (Bystritskaya, Bilichenko, 2022). The overall incidence of asthma in the Republics of Khakassia and Tyva proved to be consistent with national trends; however, precise data were not provided by the available official sources. According to media reports, approximately 6,500 cases of asthma have been registered in the Republic of Khakassia as of 2022. The Khakassia Ministry of Health has noted an increase in the prevalence of severe asthma. In the Republic of Tyva, as of 2023, there has been about 1,000 cases of asthma.

The high heritability coefficient for asthma development indicated the role of genetic factors, i. e. up to 70 % of asthma cases are caused by genetic factors, with the heritability coefficient being estimated to be up to 74 % in adults and up to 90 % in children (Hernandez-Pacheco et al., 2019). The proportion of phenotypic dispersion of a trait attributable to common single nucleotide polymorphisms, estimated within the framework of the inferred predisposition model (h2 g ), is higher for childhood asthma (aged 0 to 19 years; (h2 g ) = 25.6 %) than for asthma with the onset recorded between the ages of 20 and 60; (h2 g ) = 10.6 % (Ferreira et al., 2019). Furthermore, genome-wide association studies (GWAS) have identified more than 150 asthma risk loci, including IL13, IL33, IL1RL1, ADAM33, ORMDL3, ADRB2, FLG (Pividori et al., 2019). Ethnic specificity for the identified genetic associations is also worth noting. For example, when analyzing subgroups for ethnicity, a significant association between the rs2243250 IL4 polymorphism and asthma was found in Asians and Caucasians, but not in African Americans (Nie et al., 2013). In addition to genes encoding proinflammatory cytokines, polymorphisms in genes regulating the metabolic processes make a significant contribution to the development and control of asthma. Thus, the G allele of rs37973 GLCCI1 was associated with both uncontrolled asthma and a decrease in respiratory function indicators in Tatar population of Russia, and the T allele of rs2305089 TBXT, in the Bashkirs (Fedorova et al., 2019).

A total of 167 genes have been identified for the Russian population, with 11 polymorphic variants demonstrating a significant association with asthma. The data obtained on a large sample of the Federal Medical and Biological Agency of Russia have indicated specific genetic variants that can both increase and decrease the chance of developing the disease, which will certainly be important for the development of preventive diagnostic methods (Akhmerova et al., 2023). The distribution frequency of significant polymorphisms in European and Asian populations has not been sufficiently studied, which puts emphasis on the currently important ethnic factor for the indigenous peoples of Russia, with significant differences in genetic profile associated with liability to disease and behavior being revealed (Smolnikova, Tereshchenko, 2020; Smolnikova et al., 2022; Malarchuk, 2024; Malarchuk et al., 2024; Gusareva et al., 2025; Malarchuk, Litvinov, 2025).

Searching the PubMed database for the keywords “bronchial asthma AND gene AND ethnic group AND nationality” yielded 12,950 results over the past five years (2020–2025). Searching the eLibrary for a similar query “bronchial asthma, gene, ethnic group, nationality” for the same period yielded 125 publications, demonstrating not only research interest in this problem but also an imbalance in global populations studied, as most publications concentrate on large ethnic groups, while indigenous peoples of Russia, including those of the Angara-Yenisei region, are understudied, limiting the possibility for personalized approaches to diagnosis and treatment to be developed.

In contrast to calculating the prevalence of genotypes and alleles of isolated polymorphisms, calculating haplotypes of polymorphisms of asthma-predisposing genes is important for identifying synergistic effects, since protein function is often affected by the allele combinations in a haplotype in a nonadditive manner (Belyaeva, 2020), as well as for personalizing therapy, since taking into account the haplotype allows one to optimize the prediction of treatment response (Ouyang, Krontiris, 2006). In addition, defining haplotypes as ethnospecific risk profiles allows for characterization of regional features of morbidity and disease prevention (Tereshchenko, Smolnikova, 2020).

IL13 and ADAM33 are some of the key asthma-predisposing genes.

Interleukin-13 (IL-13) is a central mediator of immunoglobulin E (IgE) synthesis, regulates allergic inflammation, and promotes excessive mucus production, including for asthma (Gao et al., 2017). IL-13-induced periostin (Krasilnikova et al., 2020) modulates airway inflammation and remodeling (Izuhara et al., 2016), thereby promoting epithelial-mesenchymal transition and fibrosis, respectively (Sidhu et al., 2010). The risk of developing allergic asthma in Asian and Caucasian populations has been shown to raise with IL-13 overexpression (Resende et al., 2017; Smolnikova et al., 2023), but in the Saudi population, for example, no significant association was found (Halwani et al., 2018).

The minor A allele of the rs20541 polymorphism (G>A), located in exon 4 of the IL13 gene (5q31), is associated with increased total IgE levels in the serum, high serum IL-13 and periostin concentrations (Hafez et al., 2022). The significance of this polymorphism for the IL13 gene has been verified by population studies in Russia. So, in the Republic of Bashkortostan, polymorphic variants of IL13, IL4 and CCL11 have been shown to be significant risk factors associated with the development of asthma (Karunas et al., 2012). The G allele of rs20541 IL13 is more common in asthma children of Kazakh and Han (China) ethnic groups (Bai et al., 2023). Carriers of the G allele of this polymorphism respond better to combination therapy with budesonide/fluticasone propionate in the treatment of moderate to severe asthma (Liu et al., 2020). Korean children carrying the A allele of rs20541 IL13 showed increased total IgE levels and a greater maximum percent fall in FEV1 during exercise-induced bronchoconstriction (EIB) compared to GG homozygotes.

The rare T allele of the rs1295686 IL13 polymorphism is associated with a high risk of developing bronchial asthma, but the exact molecular mechanism is poorly understood (Halwani et al., 2018). In the Saudi population, the CT and TT genotypes raise the risk of asthma in a dominant inheritance pattern (Halwani et al., 2018).

The single nucleotide polymorphism (SNP) rs1800925 (C>T) is located in the promoter region of the IL13 gene. Overexpression of interleukin-13 caused by the presence of the T allele at rs1800925 causes is likely to be due to increased binding of nuclear proteins at this site (van der Pouw Kraan et al., 1999; Gaceja et al., 2023). An association between the T allele of rs1800925 IL13 and a better response to Montelukast treatment has been revealed (Kang et al., 2008).

The CTA haplotype having risk alleles of IL13 polymorphisms (rs1881457-C, rs1800925-T, rs20541-A) was shown to be more frequently found in individuals who did not respond to Montelukast (Kang et al., 2008). Carriers of the CGTG haplotype for IL13 (rs762534, rs20541, rs1295686, rs1800925) have a low risk of developing asthma in the Saudi population, whereas CATG and CATA have a higher risk, although this result is not statistically significant (Halwani et al., 2018).

The ADAM33 protein belongs to members of the metalloprotease family containing disintegrin and metalloprotease domains. It plays a pivotal role in airway remodeling due to its protease activity (breakdown of extracellular matrix proteins with an effect on bronchial structure), adhesive function (cellmatrix interactions via the disintegrin domain), and specific location in lung fibroblasts and bronchial smooth muscle cells (Camoretti-Mercado, Lockey, 2021). Changes in the activity of the ADAM33 protein encoded by the polymorphic ADAM33 gene lead to airway remodeling and a high risk of developing asthma in children (Lambrecht, Hammad, 2012; Sun et al., 2017). Furthermore, ADAM33 is involved in the activation of cytokines produced by Th2 cells (Zheng et al., 2015). Increased ADAM33 expression plays a potential role in the eosinophilic/ type 2 inflammatory endotype of asthma, considered to be the most severe and treatment-resistant disease variant (Sunadome et al., 2017). ADAM expression has been shown to increase in human fibroblasts in vitro in asthmatic patients exposed to interleukin 4 (IL-4) and IL-13 in a time- and concentrationdependent manner. In asthmatic patients, IL-4 and IL-13 can significantly increase ADAM33 mRNA expression in human fibroblasts in a concentration- and time-dependent manner (Jie et al., 2009).

The A allelic variant of the rs44707 (G>A) polymorphism of ADAM33 (20p13) alters the conformation of the disintegrin domain, breaking down the adhesive properties of the ADAM33 protein (Van Eerdewegh et al., 2002). For the Li population (China), the A allele of rs44707 ADAM33 was shown to be significantly more common among patients with asthma compared to the control group (Shen et al., 2017). The meta-analysis (4,096 asthma patients and 3,767 controls) did not reveal a significant association between the rs44707 ADAM33 polymorphism and the risk of developing asthma in the overall group and in subgroup analysis by ethnic groups. However, age-adjusted analysis demonstrated that the A allele and AA+GA genotypes are associated with asthma in children (Li et al., 2019).

The rare A allele of the rs2280090 polymorphism of ADAM33 (G>A) is associated with an increased risk of developing asthma and accelerated decline in lung function in children in China (Li et al., 2019), with this being verified in the meta-analysis (Sun et al., 2017). In the Bengali population (India), carriers of the GG genotype of rs2280090 ADAM33 and the GGA haplotype (rs2280090, rs44707, rs3918396) have an increased risk of developing asthma (Sultana et al., 2023); however, in the Punjabi population of Pakistan, no significant associations were found, whereas the strong allelic co-genetic linkage was shown in the study population (Ghani et al., 2019).

The G allele of the rs2787094 (G>C) polymorphism of ADAM33 enhances transcription factor binding and ADAM33 expression in the bronchial wall (Holgate et al., 2006; Li et al., 2019). In Chinese patients with asthma, the CG and GG genotypes of rs2787094 ADAM33 were shown to be associated with a nearly 2-fold increased risk of developing asthma in children (Qu et al., 2011) and in the Li population (Hainan Island, China) (Shen et al., 2017), with the same association being found in an Iranian cohort (Shahhosseini, Zare, 2023). However, in the Indonesian population, no association of rs2787094 with asthma was shown (Viyati et al., 2023). The meta-analysis (7,109 asthma patients and 7,794 controls) did not give a significant association between the rs2787094 ADAM33 polymorphism and asthma; however, when analyzing subgroups by ethnicity, the risk of developing asthma was shown for Caucasians, and by age, for adults (Li et al., 2019).

When analyzing haplotypes, it was shown that haplotypes encoded H1 (rs511898-T, rs2280089-G, rs2280090-G, rs2280091-A, rs2787094-G, rs612709-G), H3 (rs511898-C, rs2280089-G, rs2280090-G, rs2280091-A, rs2787094-G, rs612709-G), H5 (rs511898-T, rs2280089-A, rs2280090-G, rs2280091-A, rs2787094-C, rs612709-G) and H8 (rs511898-C, rs2280089-G, rs2280090-G, rs2280091-A, rs2787094-C, rs612709-G), containing risk alleles of the ADAM33 polymorphisms, double the risk of developing childhood asthma (China) (Qu et al., 2011).

Given the limited data on haplotypes and their role in liability to asthma in global and Russian populations, the analysis of allele combinations of genes regulating the immune response (IL13) and airway remodeling (ADAM33) is a promising area of research. In this regard, and taking into account the pronounced ethnic specificity of associations of gene polymorphisms with diseases, the aim of this study is to make a comparative analysis of haplotype combinations in the IL13 (rs20541, rs1800925, rs1295686) and ADAM33 (rs2280090, rs2787094, rs44707) genes in different ethnic groups to identify candidate population-specific genetic markers at asthma-associated loci.

## Materials and methods

In the present work, a genetic study of adolescents aged 12 to 18 years in Eastern Siberia was carried out. The nationality of the studied adolescents was verified by the nationality of both parents and was defined as Russians (n = 293), Tuvans (n = 158), and Khakassians (n = 73). Demographic data (i. e. age, gender, and nationality of parents) were taken from questionnaires collected within the framework of the State Assignment of the Scientific Research Institute of Medical Problems of the North (2018–2020).

The study object was DNA isolated from saliva. Saliva samples were collected using “Saliva DNA Collection and Preservation Devices” (Norgen Biotek, Canada). DNA extraction was performed using the DIAtomTM DNA Prep 100 reagent kit (Isogen Laboratory LLC, Russia). DNA concentration was measured using a Qubit 4.0 fluorimeter (Thermo Fisher Scientific, USA). Genotyping of IL13 (rs20541, rs1800925, rs1295686) and ADAM33 (rs2280090, rs2787094, rs44707) polymorphisms was performed by real-time polymerase chain reaction (RT-PCR) using oligonucleotide primers, fluorescently labeled TagMan probes (DNA Synthesis LLC, Russia) and RotorGene Q 6plex (QIAGEN, Germany).

The study was approved by the Ethics Committee of the Federal Research Center Krasnoyarsk Science Center of the Siberian Branch of the Russian Academy of Sciences (Protocol No. 6 dated December 6, 2012). The parents gave written informed consent for the study

The frequency of occurrence of the analyzed traits was expressed as absolute and relative values. Differences at a significance level of p < 0.05 were considered to be statistically significant. Statistical data processing was performed via the RStudio 4.3.2 software environment (Posit, USA). Genotype frequencies were analyzed using the specialized SNPassoc package, haplotype analysis and assessment of linkage disequilibrium patterns between the studied gene polymorphisms were provided using the “haplo.stats” package for R. EM analysis (Expectation-maximization) was applied with the LR calculation (Likehood Ratio Test), df (Degrees of Freedom), Dʹ (D prime, the standardized coefficient of linkage disequilibrium) and r2 (the square of the Pearson correlation coefficient). The genotype distribution across polymorphic loci was tested for compliance with Hardy–Weinberg equilibrium using the χ2 test. Comparative Population genetic analysis of the distribution of the haplotypes studied in this work with global distributions was performed with data from the 1000 Genomes Project. Reference haplotype frequencies for the IL13 and ADAM33 loci were downloaded using the LDlink (LDhap) resource (https://ldlink.nih.gov/ldhap?ref=82878) for the CEU (Utah residents from North and West Europe) and CHB (Han Chinese in Beijing, China) cohorts.

## Results

The analysis revealed the haplotype frequency distribution of the IL13 and ADAM33 polymorphic genes in Russian, Tuvan, and Khakass populations. Haplotype combination of the IL13 gene includes the rs20541, rs1295686, and rs1800925 polymorphisms, while that of the ADAM33 gene consists of the rs44707, rs2280090, and rs2787094 polymorphisms.

Data on the frequency distribution of haplotypes of the IL13 gene polymorphisms (rs20541, rs1295686, and rs1800925) are presented in Table 1. The most common haplotype in all examined groups turned out to be GCC (Russians – 37.5 %, Tuvans – 49.4 %, Khakassians – 31.5 %). This haplotype consists of wild allelic variants of the three studied polymorphisms. The ATT haplotype was the second most common in Russians (29.4 %); in Tuvans and Khakassians, it was ATC (21.5 and 31.5 %, respectively). The ATT haplotype consisting of rare allelic variants rs20541 + rs1295686 + rs1800925 of IL13 was significantly more common among Russians compared to Tuvans and Khakassians (29.4 % versus 17.1 and 23.3 %, respectively).

**Table 1.**
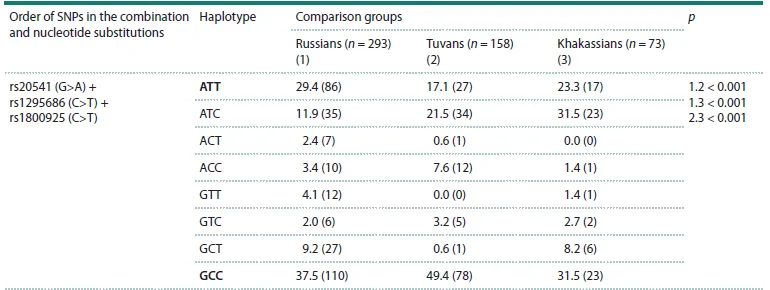
Haplotype frequency distribution for the IL13 gene (rs20541, rs1295686, and rs1800925), % (n) Note. Zero haplotype frequency values are not included in the table.

To assess the presence of linkage disequilibrium between the studied loci, EM analysis was used, and the results revealed a statistically significant presence of linkage disequilibrium between the rs1295686, rs1800925 and rs20541 IL13 polymorphisms (Table 2).

**Table 2.**
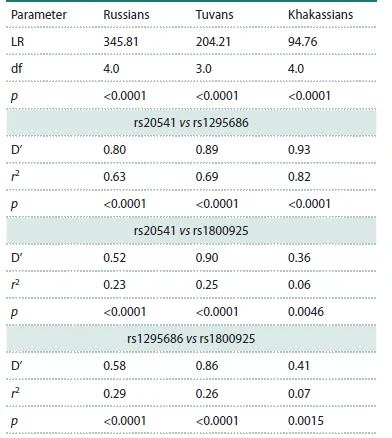
Linkage disequilibrium analysis of the IL13 gene polymorphisms in Russians, Tuvans and Khakassians

A significant deviation from linkage equilibrium was shown in all three populations (p < 0.0001), with the most pronounced general disequilibrium observed in Russians (LR = 345.81), whereas this finding was almost 3.5 times lower in Khakassians, indicating population differences in haplotype structure. The maximum interethnic differences were revealed for the rs20541 and rs1800925 polymorphisms. So, in Tuvans, a strong linkage was observed (Dʹ = 0.90), developing a stable haplotype block; this indicator was lower (Dʹ = 0.36) in Khakassians, indicating a partial loss of association between the alleles. On the other hand, the maximum Dʹ value was shown for the rs1295686 and rs1800925 pair in Tuvans (Dʹ = 0.86). The correlation coefficient (r2 = 0.82) was shown to exceed the threshold (r2 > 0.8) only in the Khakass people in the rs20541 and rs1295686 pair, which allows one to consider these polymorphisms as interchangeable research markers. In Russians and Tuvans, this indicator was at the level of moderate correlation (0.63 and 0.69, respectively), which does not allow one to completely replace one polymorphism with another. In the Khakass people, a low correlation coefficient for pairs of polymorphisms involving rs1800925 (0.06 and 0.07) was revealed, limiting the use of this locus as an interchangeable risk marker for other polymorphisms in this population. At the same time, in Tuvans, the rs1800925 polymorphism demonstrated a strong linkage with other polymorphisms, suggesting the development of a haplotype block including all the studied IL13 polymorphisms.

The haplotype frequencies of ADAM33 (rs44707, rs2280090 and rs2787094) were presented in Table 3. In Russians, the most frequent haplotypes were GGG (24.9 %) and GGC (22.2 %); however, in Tuvans and Khakassians, the most frequent was the GGC haplotype (42.4 and 35.6 %, respectively). The second most common haplotype among Tuvans and Khakassians was TGG (27.8 and 24.7 %, respectively). The TGC haplotype prevalence was lower in Russians compared to Tuvans and Khakassians (8.5 % versus 14.6 and 13.7 %, respectively). And, conversely, the TAG haplotype was more common in the Russian population compared to Tuvans and Khakassians (11.3 % versus 3.8 and 6.8 %, respectively). The GAC haplotype was significantly more common among Russians (4.8 %) compared to Khakassians and Tuvans (1.4 and 0.0 %).

**Table 3.**
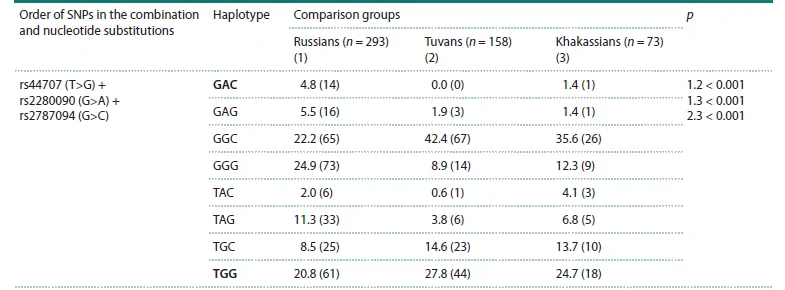
Haplotype frequency distribution for the ADAM33 gene (rs44707, rs2280090, and rs2787094), % (n) Note. Zero haplotype frequency values are not included in the table.

To assess the presence of linkage disequilibrium between the studied loci, EM analysis was used, with a statistically significant presence of linkage disequilibrium between the rs44707, rs2280090 and rs2787094 ADAM33 polymorphisms found (Table 4).

**Table 4.**
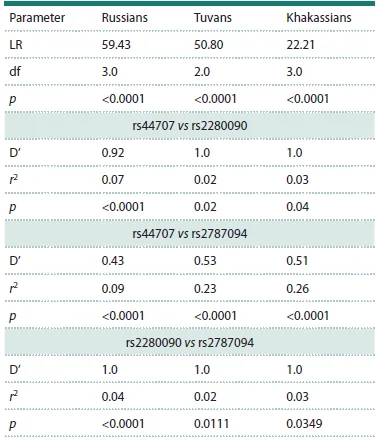
Linkage disequilibrium analysis of the ADAM33 gene polymorphisms in Russians, Tuvans and Khakassians

A significant deviation from linkage equilibrium was revealed in all the studied populations (p < 0.0001): the most pronounced general disequilibrium was observed in Russians (LR = 59.43), and in Khakassians, this indicator was almost 2.7 times lower. For the pair of rs2280090 and rs2787094 polymorphisms, a complete linkage disequilibrium (Dʹ = 1.0) was recorded for all populations; accordingly, the alleles of these loci are always inherited together, developing a stable haplotype block. Similar results were shown for the pair of rs44707 and rs2280090 polymorphisms (in Russians Dʹ = 0.92, in Tuvans and Khakassians Dʹ = 1.0). Different results were obtained for the rs44707 and rs2787094 polymorphisms, i. e. the Dʹ values, having moderate linkage, ranged from 0.43 to 0.53, respectively, pointing to partial recombination between these loci. The ratio of the Dʹ and r2 values is worth noting. Despite the complete linkage of rs2280090 and rs2787094, the r2 values were below the moderate correlation threshold (r2 > 0.33), which does not allow these risk markers to be used interchangeably in studies. The highest r2 values for the rs44707 and rs2787094 pair were found in Khakas- sians (0.26) and Tuvans (0.23), this value being much lower (0.09) in Russians, implying the population differences in the balance of allele frequencies of the ADAM33 gene polymorphisms.

The prevalence of haplotypes of IL13 and ADAM33 in samples of Russians, Tuvans, and Khakassians differed significantly from those presented for the CEU and CHB reference populations. The populations studied did not completely correspond to the European (CEU) or East Asian (CHB) profile (Table 5).

**Table 5.**
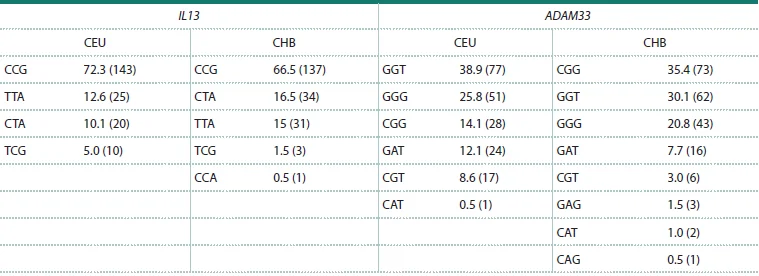
The prevalence of haplotypes of IL13 (rs20541, rs1295686 and rs1800925) and ADAM33 (rs44707, rs2280090 and rs2787094) in the European (CEU) and East Asian (CHB) populations, % (n)

## Discussion

In the current study, a comparative analysis of the prevalence of haplotypes of the IL13 (rs20541, rs1295686, and rs1800925) and ADAM33 (rs44707, rs2280090, and rs2787094) polymorphisms in Russian, Tuvan, and Khakass populations was carried out.

The “risk” haplotype ATT combines rare alleles of the rs20541-A, rs1295686-T, and rs1800925-T polymorphisms of the IL13 gene. This haplotype contains functionally significant variants that mediate chronic inflammation and lead to mucus hyperproduction (Gaceja et al., 2023). In particular, the presence of the T allele of rs20541 causes an increase in the affinity of the IL-13 ligand for the receptor, which, in turn, results in hyperproduction of IgE and periostin (Halwani et al., 2018; Hafez et al., 2022). At the same time, the T allele of rs1295686 is associated with an increased synthesis of IL-13 due to an enhancement in the transcriptional activity of the IL13 gene (Halwani et al., 2018). The presence of the T allele of rs1800925 is associated with a decreased affinity of IL-13 for the receptor and an increased IL-13 concentration in the blood (Arima et al., 2002). So, the risk haplotype ATT is characterized by a combination of increased IL13 expression and high affinity for the receptor, leading to bronchial hyperreactivity and eosinophilic inflammation.

Our data revealed statistically significant differences in the prevalence of the risk haplotype ATT among Russians (29.4 %), Tuvans (17.1 %), and Khakassians (23.3 %), which is consistent with the known population patterns in the distribution of individual alleles for IL13. So, rare alleles of the rs20541 and rs1295686 polymorphisms also predominate in the Saudi population of patients with asthma; however, their low prevalence in the general population limits the statistical power of the study (Halwani et al., 2018). In addition, the CATG and CATA haplotypes (rs762534, rs20541, rs1295686, rs1800925) of the IL13 gene, which contain the ATC and ATT haplotypes of the polymorphisms studied, demonstrate a tendency toward an increased risk of developing asthma in the Saudi population (Halwani et al., 2018). The CGTG haplotype (rs762534, rs20541, rs1295686, rs1800925) demonstrates a protective effect on the asthma development in the Saudi population (Halwani et al., 2018).

It is important to note that even though the CGTG haplotype contains a minor risk allele T of the rs1295686 polymorphism associated with airway inflammation, its combination with the G allele of rs20541 neutralizes the risk, emphasizing the epistatic effect of loci, when the effects of the T allele of rs1295686 are modulated by the G allele of rs20541. Indeed, the CTA haplotype (rs1881457, rs1800925, rs20541) of the IL13 gene in Korean children with asthma is associated with non-response to therapy with Montelukast, a leukotriene receptor antagonist (Kang et al., 2008). The key factor in this combination is the risk allele T of the rs1800925 polymorphism, which causes overexpression of IL-13. In the Chinese population, carriers of haplotypes that included both the T allele of rs20541 and the T allele of rs1800925 had very high IgE level in cord blood, predicting asthma development in childhood (Hua et al., 2021).

The Russian population has a higher frequency of haplotypes including rare alleles of the studied IL13 gene polymorphisms, which is likely to point to an increased genetic predisposition to asthma development in this group compared to Tuvans and Khakassians. Interestingly, in Khakass and Tuvans, the frequency of the ATC haplotype of the IL13 gene, which contains two of the three studied alleles associated, according to the literature, with asthma, is 2–3 times higher than in Russians. The absence of the T allele of rs1800925 in this haplotype, which is considered to be a key marker of asthma risk in some studies, suggests that the functional significance of this haplotype may be different. The problem of its contribution to disease predisposition warrants further investigation in case-control studies involving asthma patients from these ethnic groups.

The GAC haplotype (rs44707-G, rs2280090-A, rs2787094-C) of the ADAM33 gene combines rare risk alleles of the studied polymorphisms associated with changes in the structural and functional properties of the ADAM33 protein. In particular, the presence of the G allele of rs44707 leads to increased transcriptional activity and, in turn, to hyperproduction of ADAM33 in airway tissue cells (Xue et al., 2013). Therefore, the phosphorylation of the ADAM33 protein is affected by the A allele of rs2280090, changing its proteolytic activity and the ability to mediate airway remodeling. The C allele of rs2787094 is associated with increased ADAM33 expression, which facilitates bronchial smooth muscle hypertrophy (Li et al., 2019). According to our study, the risk haplotype GAC of ADAM33 is statistically significantly more common among Russians (4.8 %) compared to Khakassians (1.4 %) and Tuvans (0.0 %). These findings are consistent with the published data. Thus, in the study (Zhou et al., 2011), another haplotype H5 of the ADAM33 gene, also including rare alleles at the rs2280090 and rs44707 loci in a different combination, was associated with a 2-fold increased risk of asthma in children of the Han population of China. Given the higher frequency of the GAC haplotype identified in the Russian population, the distribution patterns of ADAM33 gene variants can be assumed to be able to contribute to the inter-ethnic differences in asthma prevalence previously noted for the population of Eastern Siberia (Konopleva et al., 2017; Afonicheva et al., 2025a).

According to our data, the GGG haplotype of the ADAM33 gene is more common in Russians (24.9 %) than in Tuvans and Khakassians (8.9 and 12.3 %). The haplotype encoded as H1 (rs2280090-G + rs2787094-G + rs44707-G) in the Northern Chinese population is associated with severe asthma in children (Qu et al., 2011). Considering that carriers of rare alleles G of the rs44707 polymorphism and A of the rs2787094 polymorphism of ADAM33 have a greater allergen sensitivity and pronounced lung function decrease (Sleziak et al., 2024; Afonicheva et al., 2025b), the risk of developing asthma exacerbations among Russians is assumed to be definitely higher than among other populations studied in the work. It should be taken into account that the low prevalence of the GGG haplotype of ADAM33 in the Tuvan and Khakass populations limits the statistical analysis power of their association with the disease.

The GGC haplotype containing two of the three studied risk variants of the ADAM33 gene polymorphisms is significantly more common in Tuvans and Khakassians than in Russians. Due to the absence of the A allele of the ADAM33 gene for rs2280090 in this haplotype, considered in the literature to be a marker of underlying risk for asthma, we hypothesize that the functional effects of the GGC haplotype may differ from those with a complete set of risk alleles. However, weather the clinical significance of this haplotype and its contribution to genetic predisposition to asthma development remains unclear.

The identified strong linkage disequilibrium between the studied IL13 and ADAM33 gene loci means that the allelic variants of the studied gene polymorphisms are inherited as haplotype blocks. Therefore, the consideration of polymorphism combinations as haplotypes has been justified. Population differences in the distribution of haplotype frequencies, indicating both the different levels of LD in Russians, Tuvans and Khakassians and the presence of genetic drift, have been demonstrated in our study.

The differences identified in the haplotype distribution of the IL13 and ADAM33 loci between our sample data and the CEU and CHB populations reveals a unique genetic structure of the studied ethnic groups

## Conclusion

The distribution patterns of IL13 and ADAM33 gene haplotypes in Russian, Tuvan, and Khakass populations were shown in the study. We identified statistically significant differences in the frequencies of haplotypes, including polymorphism alleles, which, according to the literature, are associated with a predisposition to asthma. IL13 gene haplotypes containing rare “risk” alleles were established to occur at a higher incidence in the Russian population compared to Tuvans and Khakassians. Our results also demonstrated that the risk GAC haplotype of the ADAM33 gene is significantly more common in Russians than in Khakassians and Tuvans.

The differences identified in haplotype distribution may reflect genetic characteristics of the studied populations, potentially influencing interpopulation differences in asthma prevalence. However, for this hypothesis to be validated, case-control studies in each of the studied ethnic groups are needed. The obtained data highlight the multifactorial nature of asthma and the need to take into account ethno-geographical characteristics when studying genetic susceptibility to the disease. The results and the role of the identified haplotypes in asthma development have to be verified in functional studies with larger sample sizes.

## Conflict of interest

The authors declare no conflict of interest.
